# Economic Analysis of the European Healthcare Burden of Sternal-Wound Infections Following Coronary Artery Bypass Graft

**DOI:** 10.3389/fpubh.2020.557555

**Published:** 2020-10-23

**Authors:** Maximilian Blüher, Dominique Brandt, Julie Lankiewicz, Peter J. Mallow, Rhodri Saunders

**Affiliations:** ^1^Coreva Scientific GmbH & Co. KG, Königswinter, Germany; ^2^Xavier University, Cincinnati, OH, United States; ^3^Cardinal Health, Dublin, OH, United States

**Keywords:** surgical wound infection, healthcare costs, hospital costs, length of stay, Europe

## Abstract

**Background:** Sternal wound infections (SWIs) can be some of the most complex surgical-site infections (SSIs) and pose a considerable risk following coronary artery bypass graft surgery (CABG).

**Objective:** To capture the cost burden of SWIs following CABG across European countries.

**Methods:** We modeled a standardized care pathway for CABG, starting at the point of surgery and extending to 1-year post surgery. The Markov model captures the incidence and cost of an SWI (deep or superficial SWIs). The cost burden is calculated from a hospital perspective such that the main inputs relating to costs were intensive-care-unit (ICU) and general-ward (GW) days. Outpatient care, not in the hospital setting, has no cost in this analysis. Model input parameters were taken from Eurostat and a review of published, peer-reviewed literature. European countries were included in this analysis when values for 50% of the required input parameters per country were identified. Missing data points were interpolated from available data. The robustness of results was assessed via probabilistic sensitivity analysis.

**Results:** Full required input data were available for 8 European countries; a further 18 countries had sufficient data for analysis. The median (interquartile range) for SWI incidence across the 26 countries was 3.9% (2.9–5.6%). The total burden for all 26 countries of SWIs after CABG was €170.8 million. These costs were made up of 25,751 additional ICU days, 137,588 additional GW days, and 7,704 readmissions. The mean cost of an SWI ranged from €8,924 in Poland to €21,321 in Denmark. Relative to the costs of post-CABG care without an SWI complication, the incremental cost of an SWI was highest in Greece (24.9% increase) and lowest in the UK (3.8% increase) with a median (interquartile range) of 12% (10–16%) across all 26 countries.

**Conclusions:** SWIs following CABG present a considerable burden to healthcare budgets.

## Introduction

Surgical-site infections (SSIs) can be a serious complication that may present in the days or weeks following a surgical intervention. In the 50 years since Rene Favaloro pioneered coronary artery bypass graft surgery (CABG), it has become a commonly performed procedure to improve blood flow to the heart in patients suffering from severe coronary heart disease. During CABG, the afflicted part of the coronary artery is replaced by a segment taken from another major blood vessel (the harvest site). Due to the highly invasive nature of CABG procedures, post-surgical infections are especially dangerous. SSIs linked to CABG can occur either at the harvest site or at the sternum, the latter generally being considered more detrimental to patient outcomes. Sternal-wound infections (SWIs) are further divided into superficial, deep, and organ-space SWIs.

SWIs can have a substantial negative effect on patients' quality of life, in some cases even leaving them at pre-surgical quality-of-life levels (1). For hospitals, all SSIs are of considerable concern, as many healthcare regulators consider them a quality-of-care indicator. The incidence of SSIs can thus impact hospital financing and reimbursement. In the United Kingdom (UK), avoidable infections or readmissions within 30 days of a procedure are not reimbursed (2). Outside of Europe, similar schemes are in place, including in the USA and Australia (3, 4). The pros and cons of financial penalties can be debated (5), but their presence makes treating SSIs an even more costly complication for hospitals. In settings where there is no similar penalty system in place, SSIs are a burden to healthcare systems as a whole, due to the associated increased length of stay and higher costs of care (6, 7).

In recent years, SSI rates have been going down, largely due to the introduction of antibiotic prophylaxis, which is almost ubiquitous at this point. Still, SSIs are reported to occur in roughly three percent of CABG procedures in Europe (8). The WHO states that SSIs are the second most frequent type of hospital-acquired infections in both Europe and the USA (9). From both the patient's and provider's point of view, not all SSIs are equal. The impact and rates of harvest-site infections vs. deep SWIs (DSWIs) or organ-space infection (mediastinitis) is very different, but few publications or programmes report on more than one SSI definition. Variance in reporting considerably complicates the quantification of the overall burden of SSIs.

The objective of this research is to estimate the overall burden of SWIs related to CABG in Europe. In the cost model, we differentiate between superficial SWIs (SSWIs) and DSWIs to better reflect their differences in care costs. Organ-space SWIs were not considered separately but are regarded for cost computations to be equal to DWSIs. Data on disease burden are required to start taking appropriate action on current discussions and targets for implementing value-based healthcare programmes. More specifically, data on the burden of SWIs could facilitate well-informed, value-based procurement decisions for interventions allowing for incremental improvement in SWI prevention.

## Methods

### Perspective

Eurostat collects data on 34 countries. The 27 European Union countries (EU-27) plus the UK, Iceland, Macedonia, Norway, Serbia, Switzerland, and Turkey. Our target was to utilize standardized reporting methods in the Eurostat to estimate the burden of disease in the EU-27. This could then be extrapolated to the additional reporting countries if sufficient local data were available. To provide estimates, with quantified uncertainty, a published Markov model of the CABG care pathway was adapted to a hospital-cost-only perspective (10). This perspective was taken for two reasons: firstly, purchasing decisions related to CABG are likely to be taken by hospitals, and secondly, not all European countries have a single payer responsible for both in-hospital and outpatient care. The burden of CABG-related SWIs from the hospital perspective provides a measure that can be estimated for each European country, irrespective of the type of healthcare system in place. Reporting is in line with CHEERS recommendations, with the checklist available in Supplementary Table 2.

### Settings

The choice of settings was based on the 34 countries for which EuroStat gathers and publishes data. This meant that a considerable part of the data underpinning the analysis comes from the same source, hopefully, allowing for a higher level of comparability between settings. Likewise, for consistency, country-specific unit costs provided by the WHO for the general ward (GW) were used for all included countries. These costs are comprised of components such as personnel, capital, and food costs but exclude costs of drugs and diagnostic tests. No such comprehensive source was available for intensive care unit (ICU) costs, therefore, these costs were sourced from published literature. ICU costs were required to be direct hospital costs, but the costs included and their definitions varied across publications. Details on these definitions can be found in the corresponding references provided in Supplementary Table 1. In no cases were costs based of reimbursement practices or diagnosis related group (DRG) codes.

### Model Design

A Markov model previously developed and published by two of the current authors was used as the basis of this analysis (10). It was originally designed as a cost-effectiveness model and was adapted to the current analysis by removing costs and adverse events not of interest; consolidating “home” and “care home” states to “out of hospital,” as the setting had no influence on hospital costs or readmission rates; and cutting the time horizon to a single year, most relevant to hospital purchasing.

**Figure 1:**
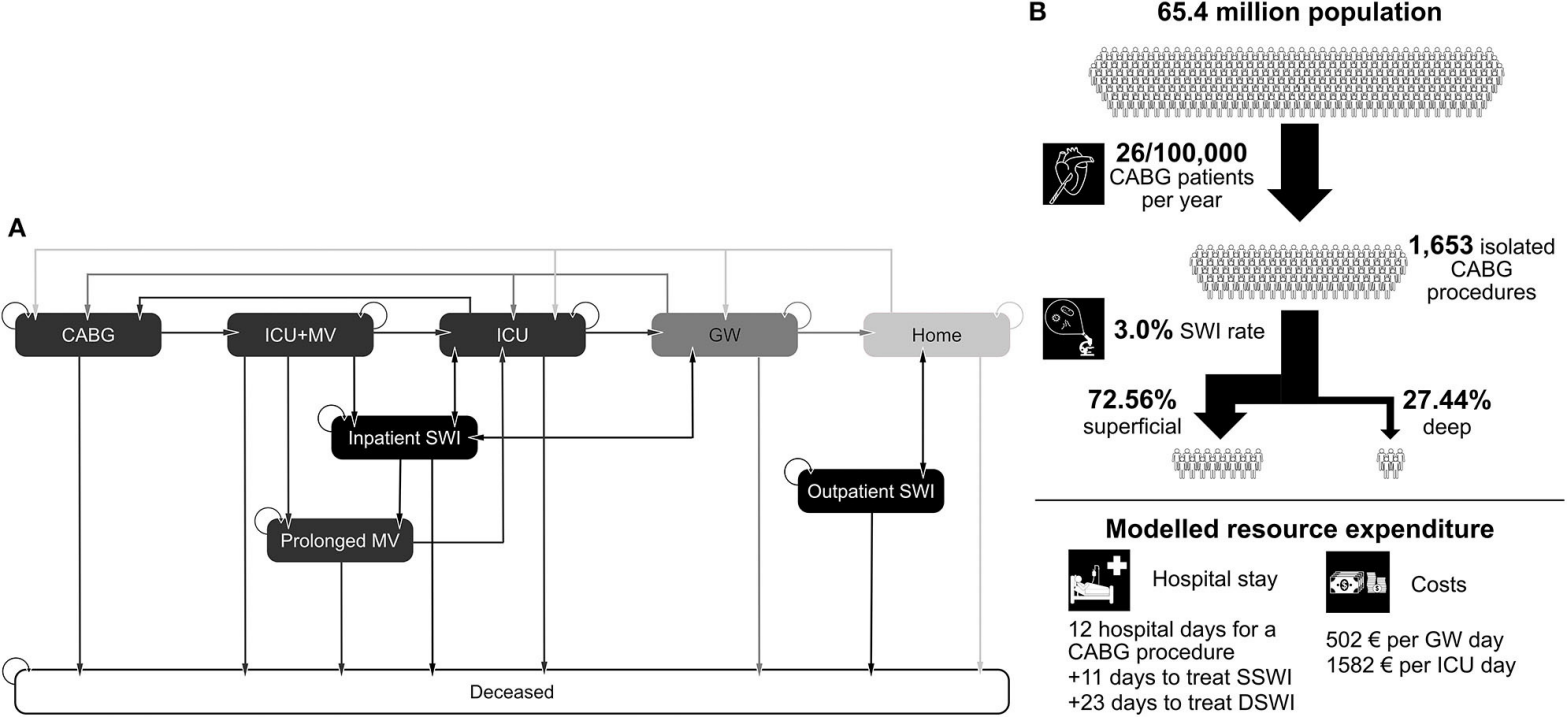
**(A)** State transitions in the Markov model. **(B)** Example of model inputs for the UK. CABG, coronary artery bypass graft; ICU, intensive care unit; GW, general ward; MV, mechanical ventilation; SWI, sternal wound infection; DSWI, deep SWI; SSWI, superficial SWI.

Patients enter the model once CABG is initiated, that is, entering the surgical suite (Figure 1A). On completion of surgery, the patient is transferred to the ICU and is receiving mechanical ventilation (MV). From this state, the patient may remain on MV, enter the prolonged MV state, or be removed from the ventilator and remain in the ICU. After exiting the ICU, patients are moved to the GW before being discharged. At any stage, and from any state, the patient can develop an SWI (either DSWI or superficial SWI—SSWI) or die. SWIs in the hospital result in additional length-of-stay and treatment costs, SWIs out of hospital are either treated in the outpatient setting (SSWIs) or require readmission (DSWIs). The incidence of SWIs is not assumed to be constant, but a cumulative incidence curve is modeled via a Dose-Response Hill curve, given data from Lankiewicz et al. (11) Both the structure of the care pathway and the shape of the cumulative incidence curve for SWIs are considered to be the same across countries. The probability of transitioning from one state to another (i.e., the speed of traversing the care pathway and the proportion of patients being in each ward) is adjusted based on country-specific data. In a similar fashion, the height of the cumulative incidence curve is adjusted per country based on the incidence of SWIs and the number of days of surveillance.

The model outputs for analysis are overall costs of care, number of ICU days, number of GW days, and number of readmissions due to SWIs.

### Model Inputs

The dataset for the model is made up of countries and inputs. For each country (*C*), there were 10 country-specific inputs (*I*) required. Let *C* = {*C*_1_, …, *C*_34_} be the set of 34 Eurostat countries and *I* = {*I*_1_, …, *I*_10_} be the set of 10 country-specific inputs. The full dataset (*X*) is thus represented by the parameters $X_{I}^{C}$ ($X_{I}^{C}\in X$) and is defined as $X_{I_{1..10}}^{C_{1..34}}=\left\{X_{I_{1}}^{C_{1}},\ldots ,X_{I_{10}}^{C_{34}}\right\}$. The data for country *C*_1_ has 10 elements and is defined as $X_{I_{1..10}}^{C_{1}}=\left\{X_{I_{1}}^{C_{1}},\ldots ,X_{I_{10}}^{C_{1}}\right\}$, whereas the data for input *I*_1_ has 34 elements and is defined as $X_{I_{1}}^{C_{1..34}}=\left\{X_{I_{1}}^{C_{1}},\ldots ,X_{I_{1}}^{C_{34}}\right\}$.

To estimate the burden of CABG-related SWIs, the following parameters were required inputs for each country in the model: the number of CABG procedures/year (included as the population size [*I*_1_] and the rate of CABG procedures per 100,000 people [*I*_2_]); the mean length of hospital stay associated with CABG (*I*_3_); the cost of an ICU day (*I*_4_); cost of a general ward day (*I*_5_); the overall SWI incidence following CABG (*I*_6_); the number of days of surveillance/follow-up (*I*_7_); the ratio of SSWI to DSWI (*I*_8_); additional length of stay to treat an SSWI (*I*_9_); and additional length of stay to treat a DSWI (*I*_10_). An example for these parameters in the UK setting can be seen in Figure 1B.

Costs were converted from local currencies to Euro where necessary. The average conversion rate for the year of the corresponding publication was used for conversion. Additionally, all costs were adjusted for inflation with country-specific inflation rates up to the year 2017.

Other parameters were required for the model to provide an estimate of burden; however, these were assumed not to be country specific (as a simplifying assumption for feasibility) and instead were held constant for each country. Examples of such input data were the costs for a readmission and the percentage of patients receiving prolonged MV. The assumption that these parameters were consistent between settings was made due to limited data availability, feasibility of data collection, and analysis of drivers of model outcomes.

During data collection, only data specifically referenced to be a DSWI, organ-space infection, or mediastinitis was associated with outcomes for DSWI in the model. General reference to an SSI or SWI was assumed to be data with relevance to a SSWI. Similarly, SSWIs and harvest-site SSIs were assumed to require the same level of treatment and costs. These assumptions were required as the necessary detail in reporting of definitions/types of SSI/SWI was not ubiquitous.

### Data Identification and Country Inclusion

Eurostat was the primary source of data. When data on required parameters was not available on Eurostat, these were identified through structured searches of PubMed and hand searches of Google scholar. Where data were not from Eurostat, peer-reviewed, published literature was a requirement. A country was included in the final analysis if there were ≥5 (out of 10 possible) country-specific data points identified.

In cases where a country had 5–9 country-specific inputs, any missing data points were interpolated from the set of countries with data. Specifically, for those countries with data, the median and interquartile range of available data were calculated. In the base-case analysis, the median value was used as the input for countries without data.

The overall robustness of model estimates to input data was assessed through a probabilistic sensitivity analysis. Here, multiple model runs were performed with a new, unique set of input parameters used for each run. Specifically, the dataset *X* ($X_{I_{1..10}}^{C_{1..34}}=\left\{X_{I_{1}}^{C_{1}},\ldots ,X_{I_{10}}^{C_{34}}\right\}$) is updated to *X'* ($X_{I_{1..10}}^{C_{1..34}}=\left\{X_{I_{1}}^{C_{1}},\ldots ,X_{I_{10}}^{C_{34}}\right\}$) each run, with new a value for every element $X_{I_{k}}^{C_{j}}$ (for country *C*_*j*_ ∈ *C* and input *I*_*K*_ ∈ *I*) being randomly drawn from between the upper and lower bound of all known input I_k_ values for all 34 countries in $X_{I_{k}}^{C_{1..34}}$. If, in the original data set X, the country-specific input value for $X_{I_{k}}^{C_{j}}$ was missing, then $X_{I_{k}}^{C_{j}}$ is assigned the randomly generated value. Otherwise, when a value for $X_{I_{k}}^{C_{j}}$ was available, $X_{I_{k}}^{C_{j}}$ is assigned the mean of $X_{I_{k}}^{C_{j}}$ and the randomly generated value. All other input parameters in the model (those that were not country specific, for example, not in *I*, were also randomly drawn for each model run. In this case, a normal distribution around the input was assumed. In total, 1,144 unique model runs were performed (44 per country). The value of 44 was arbitrary and taken because there were 44 parameters to sample each run. Results of these runs are presented as the median and range of the burden of CABG-related SWIs.

### Model Validation

During our literature review, if studies on the single-hospital burden of post-cardiac surgery SSIs/SWIs were presented, then these were tagged for potential validation and the data extracted were not used in the main model analysis. Where validation studies were identified, the hospital-specific parameters were used in the model and the burden for this hospital estimated. The results are compared to those published, allowing for readers to determine for themselves the validity of our current analysis.

## Results

**Figure 2:**
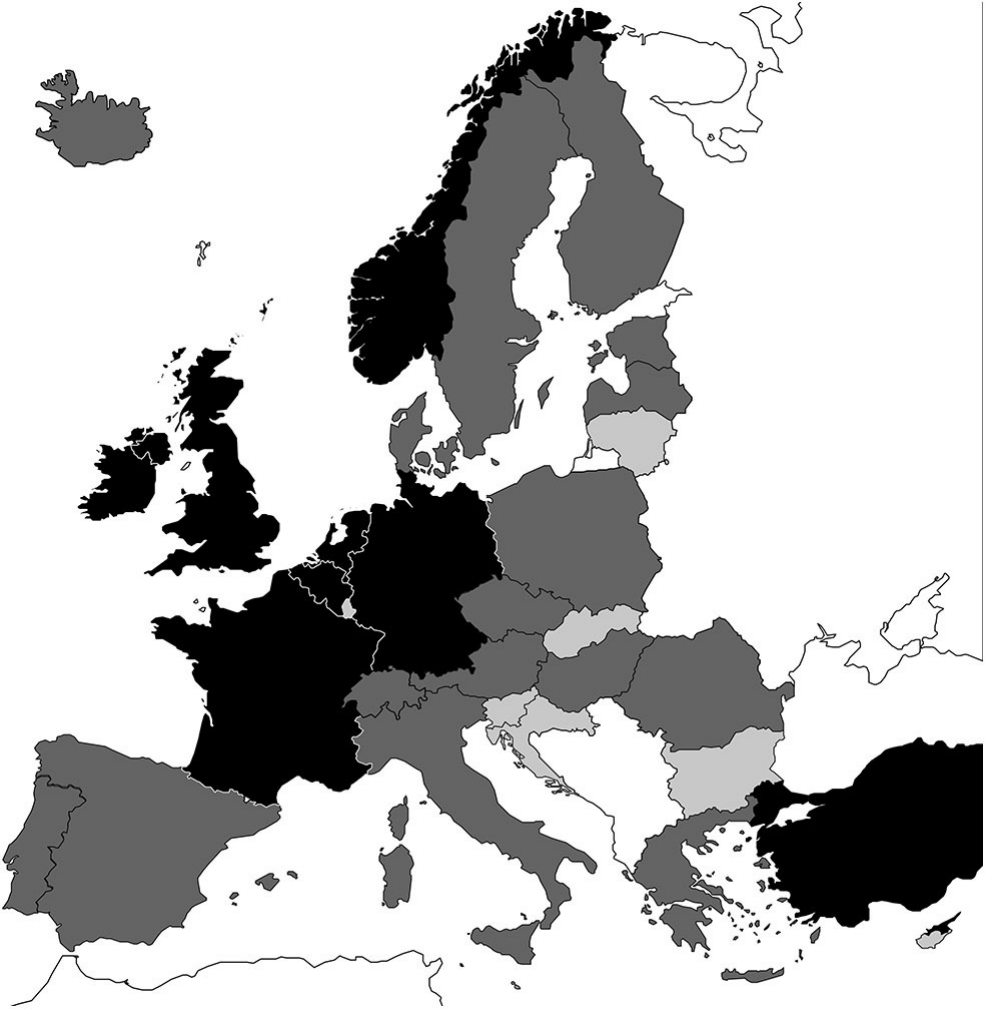
Black: full data available. Dark gray: ≥5 parameters available. Light gray: insufficient data available. White: not investigated.

Full required data were identified for 8 of our 34 target Eurostat countries: Belgium, France, Germany, Ireland, Netherlands, Norway, Turkey, and the UK. Sufficient data were identified for a further 18 countries: Austria, Czech Republic, Denmark, Estonia, Finland, Greece, Hungary, Iceland, Italy, Lithuania, Malta, Poland, Portugal, Romania, Serbia, Spain, Sweden, and Switzerland. In total, 26 countries had sufficient data (Figure 2). For the remaining eight countries, the volume of data identified was considered insufficient to provide a credible estimation of the burden. The full, tabulated and referenced dataset is provided in the Supplementary Table 1. For all other model inputs and details on the model structure please see Saunders et al. (10).

### Burden of Disease

**Figure 3:**
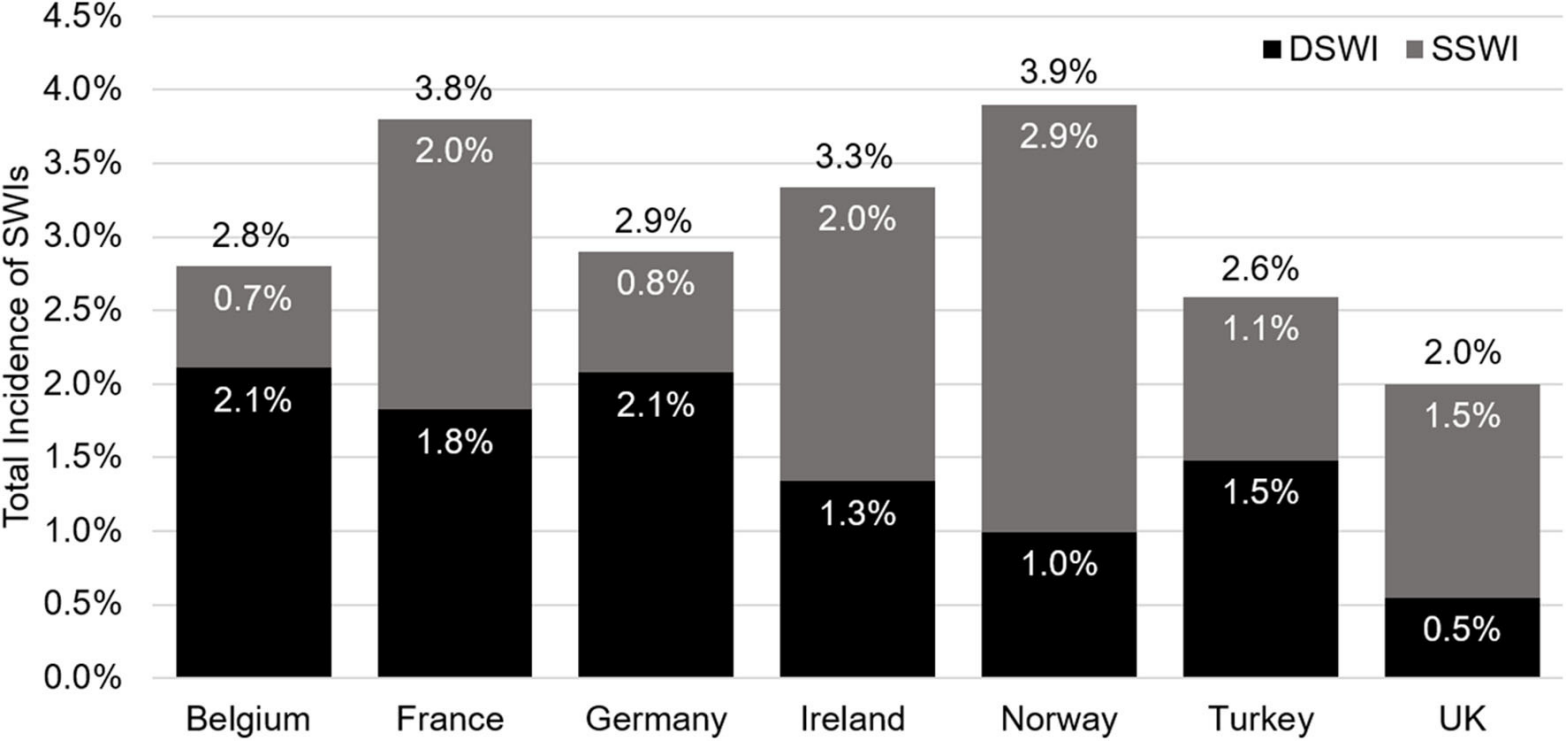
Data for the Netherlands is not depicted as this was the only country not reporting data at 30 days. The overall incidence of sternal wound infections is shown in black text above each bar, in white text are the values for DSWI (black bars), and superficial sternal wound infections (SSWI, gray bars). SWI, sternal wound infection; DSWI, deep SWI; SSWI, superficial SWI.

From a clinical perspective, the SSWI and DSWI rates are the key parameters of interest. Across the 26 countries analyzed, variation in the absolute and relative rates of SSWI and DSWIs was observed. The lowest reported prevalence of sternal wound infections was 1.6% after 30 days (Malta), with the highest being 10.4% after 6 months (Netherlands). The median (interquartile range) incidence of SWIs was 3.9% (2.9–5.6%). Of patients with a sternal wound infection, Iceland had the lowest percentage of these as DSWIs (23.3%), whereas Hungary had the highest (76%). For countries with full data, the range in SWI prevalence at 30-days was from 2.0 (UK) to 3.9% (Norway, Figure 3). In this subset of countries, Norway had the lowest ratio of SSWI to DSWIs (25.5%).

### Costs of Illness

**Figure 4:**
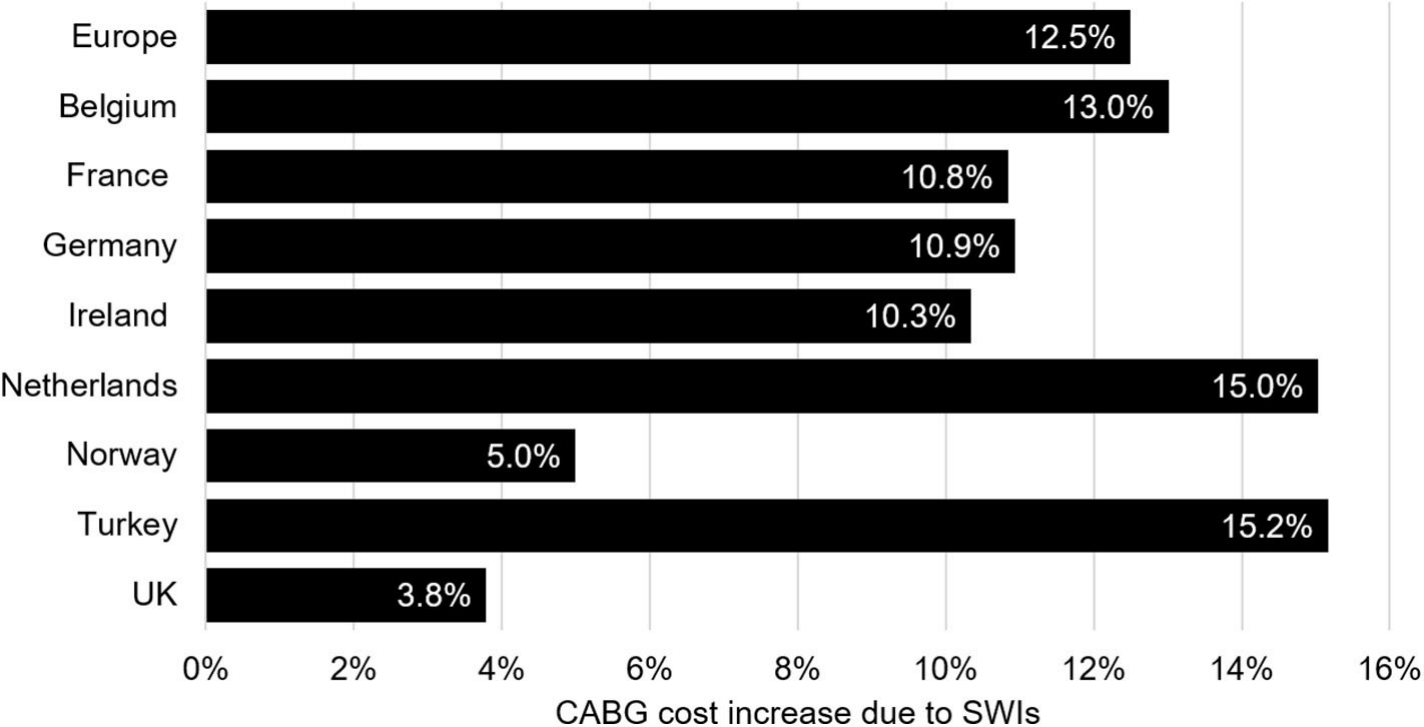
This figure contains data for the eight countries with a full set of parameters and the combined data for all countries included in the analysis (under Europe). Values represent the relative increase of CABG care costs due to SWIs. CABG, coronary artery bypass graft; SWI, sternal wound infection.

The total annual burden of SWIs after CABG for the 26 countries with sufficient data was €170.8 million. These costs are a made up of 25,751 additional ICU days, 137,588 additional GW days, and 7,704 readmissions. The mean cost of an SWI ranged from €8,924 in Poland to €21,321 in Denmark. The country with the highest cost burden was Germany, where each year €31.7 million is lost to SWI following CABG. The lowest cost burden was found in Malta, being €54,410 per year. These results, though, are likely driven by population size. Normalized by the number of CABG procedures, the highest cost burden was found in the Netherlands, with the lowest being in the UK. As healthcare costs vary by country, the cost burden may be most comparable as percentage of overall costs. Relative to the costs of post-CABG care without a SWI complication, the current cost of CABG care was highest in Greece (24.9% increase) and lowest in the UK (3.8% increase). The median (interquartile range) was 12% (10–16%). Among countries with full data, the largest increase was seen for Turkey (15.2%, Figure 4). The burden of CABG related SWIs for all countries is provided in Supplementary Table 3.

### Sensitivity Analysis

**Figure 5:**
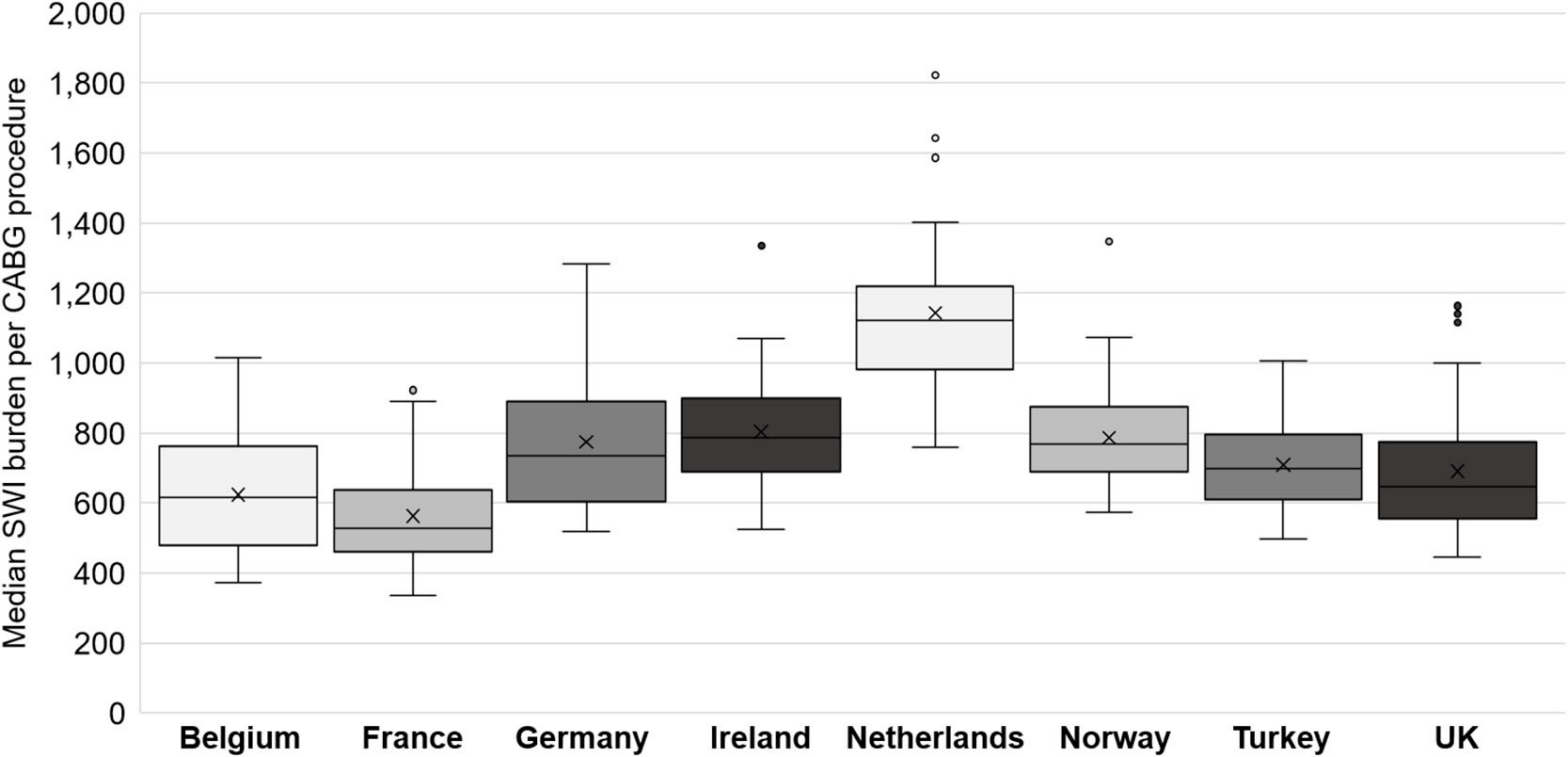
Box and whisker chart showing the median and interquartile range (IQR, central box), the mean (cross x). Each whisker extends 1.5x the IQR, points beyond this are considered outliers. CABG, coronary artery bypass graft; SWI, sternal wound infection.

The probabilistic sensitivity analysis for the 26 countries with sufficient data revealed a median (range) overall SWI burden of €181.7 million (€108.8 million to €297.8 million). The results by country, normalized by the number of CABG procedures per year, are shown for the eight countries with full data (Figure 5). Europe-wide, the burden of CABG-related SWIs was found to include a median (range) of 26,004 (12,705 to 43,264) additional ICU days; 140,448 (58,746 to 263,501) additional GW days, and 8,183 (5,173 to 12,241) avoidable readmissions. The results of the sensitivity analysis for each country are provided in Supplementary Table 4.

### Validation

Two papers were identified allowing model outcomes to be assessed for validity. In 2018, Findeisen et al. reported on the cost of post-CABG SWIs at the Jena University Hospital, Germany (7). From 983 analyzed patients, there were 126 reported SWIs over 60 days surveillance of which 96 were SSWIs. An SSWI did not increase the length of stay (14.4 days) but a DSWI resulted in 37 extra days in hospital (weighted average of DSWI and mediastinitis) (7). The cost per day in the cardiac ward was €1,010; (7) other model parameters were maintained as per the base-case model. Our model estimate for this German hospital was a burden of €7,860 per SWI, including 1,178 additional hospital days. This is very closely aligned to the burden presented by Findeisen et al.: 1,178.1 days and €7,051 (payer burden) to €8,342 (provider burden) (7). In the UK, Rochon et al. reported that 1.76% of CABG patients (mean age 65.4 years, 77% with diabetes, 9.77 days in hospital) were readmitted due to a SWI (12). Updating length of stay and patient population risk factors in our model, we calculate the percentage of patients being readmitted with a DSWI in this UK model to be 1.54%.

## Discussion

While SSI rates have reduced in recent years, our analysis suggests that the burden of SWIs on European healthcare systems remains considerable. In countries analyzed, SWIs generally increased the cost of post-CABG care by between 10 and 16%. This is a considerable increase when, in general, only 2.9 to 5.6% of procedures are complicated by a SWI. There is clearly still a need to reduce the SWI burden and, if possible, improve SWI care in cases that still occur. There are examples of hospitals implementing the former (13–15), and the impact of such a reform is significant. In the UK, implementing surveillance methods and improved infection control enabled Chiwera et al. to reduce the SSI rate for CABG from 6.5% in 2009 to 1.7% in 2016 (*p* < 0.001) (13). In the US, Kles et al. reported having attained a 0% DSWI rate for an extended period of time by implementing measures such as disposable electrocardiogram leads and pacing wires, antibiotic-coated sutures, and silver-impregnated midsternal dressings (15). In this instance, the authors reported cost savings of over $600,000 over ~ 1.5 years (15).

Innovative approaches to reduce the current burden of SSIs are likely to be of benefit to both patients and providers. Although new technology can increase the direct per-procedure cost, this may pay for itself (at least partially) if the cost of care is reduced through fewer SWIs. This is especially true if there are systems in place that “punish” hospitals for avoidable SSIs, such as in the UK, where hospitals are not reimbursed for avoidable readmissions within 30 days (2). Areas where upfront costs may result in downstream savings are of potential interest for value-based purchasing agreements. Under which, hospitals can limit their cost outlay until proof of benefit is determined. This can be advantageous to hospitals in times of constricting budgets, but also be beneficial to suppliers who can use hospital data to justify pricing based on value rather than perception.

Our study provides a first estimate of the cost burden of SWIs in Europe and gives insight into the additional cost-of-care per patient associated with SWIs. Initial comparison to a published costing study suggests that these data are a reasonable estimate for the SWI burden. Results, therefore, could be used as an initial starting point for hospitals and quality-improvement teams considering budgets for improved infection control. In doing this, readers must note the limitations of the analysis. Firstly, reporting and definitions of SSIs and SWIs were not consistent between studies. Secondly, results are from a computational model, which cannot recreate all the complexities of real-life healthcare. Some of the necessary simplifications were the assumption of an identical care pathway across all settings, a single, universal readmission cost, no inter-hospital variation, and no adjustment for patient-level risk factors. As such, results for individual hospitals will likely vary to some degree from those published here. Still, when the model was adjusted to specific published data for individual hospitals, a good fit between model outcomes and published burden was achieved.

While most simplifications were needed to create a functional model that could be used across multiple countries, the issue of limited data availability could be improved. During our search for data on this topic, Eurostat was a helpful starting point, providing some general healthcare data for all settings. When looking for more detailed information such as additional length of stay due to SWIs, or the ratio of DSWIs to SSWIs, there was a paucity of published literature. This lack of data necessitated extrapolation based on available data for several settings, which undoubtedly adds more uncertainty to the results. We encourage those working in the field of infection control to engage in surveillance and to publish and communicate their findings.

## Conclusion

SWIs, and DSWI in particular, remain a considerable problem post CABG. The treatment, additional length of hospital stay, and readmissions associated with SWIs resulted in increased cost of care—generally adding between 10 and 16% to the cost of every CABG. New interventions to further reducing SWIs would likely be of benefit to both patient outcomes and healthcare budgets. As healthcare budgets come under increasing pressure, this may be an opportunity to consider value-based purchasing schemes to offset any risk to hospitals.

## Data Availability Statement

Publicly available datasets were analyzed in this study. This data can be found at: https://ec.europa.eu/eurostat/de/home. All extracted data used in the analysis is included in the Supplementary Material.

## Author Contributions

JL and RS conceived the research project. MB, RS, and JL designed the literature searches. Literature review, analyses, and manuscript draft in full was performed by MB and RS. PM and DB reviewed data extracted and updates to the previously published health-economic model. JL, DB, and PM all provided substantial review of the manuscript. Result review and interpretation and manuscript outline approved by all authors. All authors approved the manuscript for submission and publication.

## Conflict of Interest

RS is the owner and MB is an employee of Coreva Scientific, which received consultancy fees for this work. While working on this project JL was an employee of Cardinal Health (the funder) and is now an employee of Bose. Neither Xavier University nor its affiliated authors received any compensation for participating in this research project. The remaining authors declare that the research was conducted in the absence of any commercial or financial relationships that could be construed a potential conflict of interest.
