# Supplementary material for: Economic Analysis of the European Healthcare Burden of Sternal-Wound Infections Following Coronary Artery Bypass Graft
Source: Front Public Health. 2020 Oct 23;8:557555. doi: 10.3389/fpubh.2020.557555 (PMC7645249; doi:10.3389/fpubh.2020.557555)
Supplement: Supplementary file 1 [file Table_1.docx]

Supplementary Material

# Supplementary table 1

| Country | Population | CABG rate | LoS | ICU cost | GW cost | SSI rate | % DSWI | SSI LoS | DSWI LoS |
| --- | --- | --- | --- | --- | --- | --- | --- | --- | --- |
| Name | 1,00,000s | Per 100,000 | Days | Euro per day | Euro per day | % | % of SSIs | Days | Days |
| Austria | 8.7[1] | 39.9[2] | 9.1[2] | 2,112[3] | 506[4] | 2.3[5] | 64.0[6] | 10[7] |  |
| Belgium | 11.3[1] | 63.2[2] | 5.6[2] | 706[8] | 470[4] | 2.8[9] | 75.3[9] | 13[9] | 39[9] |
| Czechia | 10.6[1] | 44.7[2] | 15.4[2] |  | 186[4] |  |  |  | 19[10] |
| Denmark | 5.7[1] | 64.8[2] | 9.2[2] | 3,382[3] | 611[4] | 3.0[11] | 50.0[11] |  | 35[12] |
| Estonia | 1.3[1] | 33.6[2] |  |  | 216[4] | 3.0[5] |  |  |  |
| Finland | 5.5[1] | 34.1[2] | 10.0[2] |  | 492[4] | 4.7[13] | 60.26[13] | 7[13] | 13[14] |
| France | 66.8[1] | 28.9[2] | 7.0[2] | 1,442[15] | 411[4] | 3.8[5] | 48.0[16] | 9[16] | 22[16] |
| Germany | 82.2[1] | 61.4[2] | 10.3[2] | 1,129[3] | 427[4] | 2.9[5] | 71.6[17] | 10[7] | 24[18] |
| Greece | 10.8[1] | 50.2 [19] |  | 572[20] | 273[4] | 5.62[21] | 61.2[22] |  |  |
| Hungary | 9.8[1] | 27.5[2] | 27.8[2] |  | 123[4] | 3.4[5] | 76[5,23] |  | 30[23] |
| Iceland | 0.3[1] | 65.1[19] | 9.9[2] |  | 616[4] | 7.3[24] | 23.3[24] | 6[24] | 43[25] |
| Ireland | 4.7[1] | 19.7[2] | 9.0[2] | 2,210[3] | 532[4] | 3.3[26] | 40.1[26] | 10[26] | 50[26] |
| Italy | 60.7[1] | 34.5[2] | 8.5[2] | 1,422[27] | 360[4] | 5.5[5] | 48.0[28] |  | 30.4[29] |
| Lithuania | 2.9[1] | 63.0[2] | 9.0[2] |  | 170[4] | 7.4[5] |  |  |  |
| Malta | 0.4[1] | 42.7[2] | 17.8[2] |  | 222[4] | 1.6[5] |  |  |  |
| Netherlands | 17.0[1] | 57.0[2] | 17.8[2] | 1,333[3] | 542[4] | 10.4[30] | 33.0[30] | 18[30] | 37[30] |
| Norway | 5.2[1] | 28.8[2] | 5.4[2] | 3,980[31] | 889[4] | 3.9[5] | 25.5[32] | 7 [33] | 22[33] |
| Poland | 38.0[1] | 51.3[2] | 7.1[2] |  | 103[4] | 2.88[34] | 62.5[34] | 12[34] | 15[35] |
| Portugal | 10.3[1] | 35.4[2] | 13.7[2] | 618[36] | 210[4] | 5.4[5] |  |  |  |
| Romania | 19.8[1] | 22.8[2] | 8.7[2] |  | 69[4] | 6.7[37] | 23.4[38] |  |  |
| Serbia | 7.0[1] | 69.9[2] | 8.2[2] |  | 36[4] | 5.7[39] | 50.0[39] | 14[40] |  |
| Spain | 46.4[1] | 17.9[2] | 11.1[2] | 1,719[3] | 325[4] | 5.9[41] | 52.8[42] | 2[32] |  |
| Sweden | 9.9[1] | 29.7[2] | 7.2[2] | 2,030[43] | 497[4] | 4.3[44] | 53.5[44] |  |  |
| Switzerland | 8.3[1] | 29.8[45] | 8.8[2] | 1,710[46] | 845[4] | 5.1[47] | 63.0[47] | 12 [48] |  |
| Turkey | 80.8[1] | 83.8 [19] | 5.5[2] | 763[49] | 271[4] | 2.6[50] | 56.6[50] | 21[51] | 35[51] |
| UK | 65.4[1] | 25.3[2] | 11.9[2] | 1,582[3] | 502[4] | 2.0[5] | 27.4[52] | 11[53] | 23[53] |

**Supplementary Table 1.** Parameters identified for each investigated country. Empty cells mark a lack of published data.

# References

1. Eurostat: Population statistics. https://ec.europa.eu/eurostat/tgm/table.do?tab=table&init=1&language=en&pcode=tps00001&plugin=1 (2019)

2. Eurostat: Cardiovascular diseases statistics. https://ec.europa.eu/eurostat/statistics-explained/index.php/Cardiovascular_diseases_statistics#Cardiovascular_healthcare (2016)

3. Bittner ,M.I., Donnelly ,M., van Zanten ,A.R.H., Andersen ,J.S., Guidet ,B., Cabello ,J.J.T., et al.: How is intensive care reimbursed? A review of eight European countries. Ann Intensive Care. 3(1):1–9 (2013)

4. World Health Organization ,(WHO): Country-specific unit costs. https://www.who.int/choice/country/country_specific/en/ (2008)

5. European Centre for Disease Prevention and Control: Healthcare-associated infections: surgical site infections. ECDC Annu Epidemiol Rep 2016 Stock ECDC. (May)(2018)

6. Andreas ,M., Muckenhuber ,M., Hutschala ,D., Kocher ,A., Thalhammer ,F., Vogt ,P., et al.: Direct sternal administration of Vancomycin and Gentamicin during closure prevents wound infection. Interact Cardiovasc Thorac Surg. 25(1):6–10 (2017)

7. Gorlitzer ,M., Wagner ,F., Pfeiffer ,S., Folkmann ,S., Meinhart ,J., Fischlein ,T., et al.: Prevention of sternal wound complications after sternotomy: Results of a large prospective randomized multicentre trial. Interact Cardiovasc Thorac Surg. 17(3):515–22 (2013)

8. Povero ,M., Pradelli ,L.: Comparison between traditional and goal directed perfusion in cardiopulmonary by-pass. A differential cost analysis in US. Farmeconomia Heal Econ Ther pathways. 16(3):77–86 (2015)

9. Poncelet ,A.J., Lengele ,B., Delaere ,B., Zech ,F., Glineur ,D., Funken ,J.C., et al.: Algorithm for primary closure in sternal wound infection: a single institution 10-year experience. Eur J Cardio-thoracic Surg. 33(2):232–8 (2008)

10. Nishimura ,K., Nakamura ,Y., Harada ,S., Saiki ,M., Marumoto ,A., Kanaoka ,Y., et al.: Vacuum-assisted closure therapy for the treatment of sternal wound infection after cardiac surgery. Kyobu Geka. 62(12):1053–5 (2009)

11. Mikkelsen ,M.M., Andersen ,N.H., Christensen ,T.D., Hansen ,T.K., Eiskjaer ,H., Gjedsted ,J., et al.: Microalbuminuria is associated with high adverse event rate following cardiac surgery. Eur J Cardio-thoracic Surg. 39(6):932–8 http://dx.doi.org/10.1016/j.ejcts.2010.09.043 (2011)

12. Juhl ,A.A., Hody ,S., Videbaek ,T.S., Damsgaard ,T.E., Nielsen ,P.H.: Deep Sternal Wound Infection after Open-Heart Surgery: A 13-Year Single Institution Analysis. Ann Thorac Cardiovasc Surg. 23(2):76–82 http://www.ncbi.nlm.nih.gov/pubmed/28163297 (2017) Accessed 2017 Oct 6

13. Järvelä ,K.M., Khan ,N.K., Loisa ,E.L., Sutinen ,J.A., Laurikka ,J.O., Khan ,J.A.: Hyperglycemic Episodes Are Associated With Postoperative Infections After Cardiac Surgery. Scand J Surg. 107(2):138–44 (2018)

14. Eklund ,A.M., Lyytikäinen ,O., Klemets ,P., Huotari ,K., Anttila ,V.J., Werkkala ,K.A., et al.: Mediastinitis After More Than 10,000 Cardiac Surgical Procedures. Ann Thorac Surg. 82(5):1784–9 (2006)

15. Lefrant ,J.-Y., Garrigues ,B., Pribil ,C., Bardoulat ,I., Courtial ,F., Maurel ,F., et al.: The daily cost of ICU patients: A micro-costing study in 23 French Intensive Care Units. Anaesthesia, Crit care pain Med. 34(3):151–7 http://www.ncbi.nlm.nih.gov/pubmed/25986476 (2015)

16. Cossin ,S., Malavaud ,S., Jarno ,P., Giard ,M., L’Hériteau ,F., Simon ,L., et al.: Surgical site infection after valvular or coronary artery bypass surgery: 2008-2011 French SSI national ISO-RAISIN surveillance. J Hosp Infect. 91(3):225–30 http://dx.doi.org/10.1016/j.jhin.2015.07.001 (2015)

17. KISS Hospital Infection Surveillance System (. (January 2005)(2010)

18. Graf ,K., Ott ,E., Vonberg ,R.P., Kuehn ,C., Haverich ,A., Chaberny ,I.F.: Economic aspects of deep sternal wound infections. Eur J Cardio-thoracic Surg. 37(4):893–6 (2010)

19. Timmis ,A., Townsend ,N., Gale ,C., Grobbee ,R., Maniadakis ,N., Flather ,M., et al.: European Society of Cardiology: Cardiovascular disease statistics 2017. Eur Heart J. 39(7):508–77 (2018)

20. Armaganidis ,A., Nanas ,S., Antoniadou ,E., Mandragos ,K., Liakou ,K., Koutsoukou ,A., et al.: Clinical factors affecting costs in patients receiving systemic antifungal therapy in intensive care units in Greece: Results from the ESTIMATOR study. Mycoses. 60(7):454–61 (2017)

21. Pitsiou ,G., Kioumis ,I., Zarogoulidis ,K., Lazaridis ,G., Papaiwannou ,A., Tsirgogianni ,K., et al.: Prophylactic antibiotic administration for post cardiothoracic surgery sternal wounds: A retrospective study. Ann Transl Med. 3(4):1–7 (2015)

22. Molnar ,L., Berhes ,M., Papp ,L., Nemeth ,N., Fulesdi ,B.: Cerebral autoregulation testing in a porcine model of intravenously administrated E. coli induced fulminant sepsis. Crit Care. 19(Suppl 1):P1 (2015)

23. Rashed ,A., Gombocz ,K., Alotti ,N., Verzar ,Z.: Is sternal rewiring mandatory in surgical treatment of deep sternal wound infections? J Thorac Dis. 10(4):2412–9 (2018)

24. Vesteinsdottir ,E., Helgason ,K.O., Sverrisson ,K.O., Gudlaugsson ,O., Karason ,S.: Infections and outcomes after cardiac surgery—The impact of outbreaks traced to transesophageal echocardiography probes. Acta Anaesthesiol Scand. 63(7):871–8 (2019)

25. Steingrimsson ,S., Gottfredsson ,M., Gudmundsdottir ,I., Sjögren ,J., Gudbjartsson ,T.: Negative-pressure wound therapy for deep sternal wound infections reduces the rate of surgical interventions for early re-infections. Interact Cardiovasc Thorac Surg. 15(3):406–10 (2012)

26. Parissis ,H., Al-Alao ,B., Soo ,A., Orr ,D., Young ,V.: Risk analysis and outcome of mediastinal wound and deep mediastinal wound infections with specific emphasis to omental transposition. J Cardiothorac Surg. 6(1):2–9 (2011)

27. Tan ,S.S., Bakker ,J., Hoogendoorn ,M.E., Kapila ,A., Martin ,J., Pezzi ,A., et al.: Direct cost analysis of intensive care unit stay in four European countries: applying a standardized costing methodology. Value Health. 15(1):81–6 http://linkinghub.elsevier.com/retrieve/pii/S1098301511035091 (2012) Accessed 2017 Mar 22

28. De Santo ,L.S., Bancone ,C., Santarpino ,G., Romano ,G., De Feo ,M., Scardone ,M., et al.: Microbiologically documented nosocomial infections after cardiac surgery: an 18-month prospective tertiary care centre report. Eur J Cardio-thoracic Surg. 33(4):666–72 (2008)

29. D’Agostino ,D., Man ,A., Santacroce ,L.: Current trends in cardiac surgery: Clinical experience in the treatment of mediastinitis with sternal wound infection through negative pressure therapy. Acta Medica Mediterr. 32(5):195–201 (2016)

30. van der Slegt ,J., van der Laan ,L., Veen ,E.J., Hendriks ,Y., Romme ,J., Kluytmans ,J.: Implementation of a Bundle of Care to Reduce Surgical Site Infections in Patients Undergoing Vascular Surgery. PLoS One. 8(8):1–7 (2013)

31. Lindemark ,F., Haaland ,Ø.A., Kvåle ,R., Flaatten ,H., Norheim ,O.F., Johansson ,K.A.: Costs and expected gain in lifetime health from intensive care versus general ward care of 30,712 individual patients: A distribution-weighted cost-effectiveness analysis. Crit Care. 21(1)(2017)

32. Figuerola-Tejerina ,A., Rodríguez-Caravaca ,G., Bustamante-Munguira ,J., María San Román-Montero ,J., Durán-Poveda ,M.: Epidemiological Surveillance of Surgical Site Infection and its Risk Factors in Cardiac Surgery: A Prospective Cohort Study. Rev Española Cardiol (English Ed. 69(9):842–8 (2016)

33. Berg ,T.C., Kjørstad ,K.E., Akselsen ,P.E., Seim ,B.E., Løwer ,H.L., Stenvik ,M.N., et al.: National surveillance of surgical site infections after coronary artery bypass grafting in Norway: Incidence and risk factors. Eur J Cardio-thoracic Surg. 40(6):1291–7 http://dx.doi.org/10.1016/j.ejcts.2011.02.038 (2011)

34. Dubiel ,G., Rogoziński ,P., Zaloudik ,E., Bruliński ,K., Rózańska ,A., Wójkowska-Mach ,J.: Identifying the Infection Control Areas Requiring Modifications in Thoracic Surgery Units: Results of a Two-Year Surveillance of Surgical Site Infections in Hospitals in Southern Poland. Surg Infect (Larchmt). 18(7):820–6 (2017)

35. Kotnis-Gaska ,A., Mazur ,P., Olechowska-Jarzab ,A., Stanisz ,A., Bulanda ,M., Undas ,A.: Sternal wound infections following cardiac surgery and their management: A single-centre study from the years 2016-2017. Kardiochirurgia i Torakochirurgia Pol. 15(2):79–85 (2018)

36. Fiorentino ,F.: Hospital-acquired infections: a cost estimation for BSI in Portugal. http://www3.eeg.uminho.pt/economia/nipe/PEJ2014/Francesca Fiorentino.pdf (2014)

37. ECDC: Anual epidemiological report for 2014: Surgical site infections. Stockholm:ECDC. (April):4–11 https://ecdc.europa.eu/en/publications-data/surgical-site-infections-annual-epidemiological-report-2016-2014-data (2016)

38. Tinica ,G., Chistol ,R.O., Enache ,M., Constantin ,M.M.L., Ciocoiu ,M., Furnica ,C.: Long-term graft patency after coronary artery bypass grafting: Effects of morphological and pathophysiological factors. Anatol J Cardiol. 20(5):275–82 (2018)

39. Živković ,I., Vuković ,P., Krasić ,S., Milutinović ,A., Milić ,D., Milojević ,P.: MINIMALLY INVASIVE AORTIC VALVE REPLACEMENT VS AORTIC VALVE REPLACEMENT THROUGH MEDIAL STERNOTOMY: PROSPECTIVE RANDOMIZED STUDY. 58(2):97–102 (2019)

40. Suljagić ,V., Jevtic ,M., Djordjevic ,B., Jovelic ,A.: Surgical site infections in a tertiary health care center: Prospective cohort study. Surg Today. 40(8):763–71 (2010)

41. ECDC: Surveillance of surgical site infections in Europe. 2013.

42. Tamayo ,E., Gualis ,J., Flórez ,S., Castrodeza ,J., Eiros Bouza ,J.M., Álvarez ,F.J.: Comparative study of single-dose and 24-hour multiple-dose antibiotic prophylaxis for cardiac surgery. J Thorac Cardiovasc Surg. 136(6):1522–7 (2008)

43. SRVN: Regionala priser och ersättningar för södra sjukvårdsregionen 2015. (2015)

44. Friberg ,Ö.: Local collagen-gentamicin for prevention of sternal wound infections: The LOGIP trial. Apmis. 115(9):1016–21 (2007)

45. Lafortune Balestat,G., Durand A. ,G.: Comparing activites and performance of the hospital sector in Europe:how many surgical procedures perdormed as inpatient and day cases? OECD, Dir Employment, Labour Soc Aff. (December)(2012)

46. Berger ,M.M., Davadant ,M., Marin ,C., Wasserfallen ,J.B., Pinget ,C., Maravic ,P., et al.: Impact of a pain protocol including hypnosis in major burns. Burns. 36(5):639–46 (2010)

47. Troillet ,N., Aghayev ,E., Eisenring ,M., Widmer ,A.F.: First Results of the Swiss National Surgical Site Infection Surveillance Program : Who Seeks Shall Find. 38(6)(2017)

48. Meszaros ,K., Fuehrer ,U., Grogg ,S., Sodeck ,G., Czerny ,M., Marschall ,J., et al.: Risk Factors for Sternal Wound Infection After Open Heart Operations Vary According to Type of Operation. Ann Thorac Surg. 101(4):1418–25 http://dx.doi.org/10.1016/j.athoracsur.2015.09.010 (2016)

49. Aygencel ,G., Turkoglu ,M.: Characteristics, Outcomes and Costs of Prolonged Stay ICU Patients. Dahili ve Cerrahi Bilim Yoğun Bakım Dergisi/ Turkish J Med Surg Intensive Care. 2(3):53–8 (2011)

50. Yavuz ,S.Ş., Tarçin ,Ö., Ada ,S., Dinçer ,F., Toraman ,S., Birbudak ,S., et al.: Incidence, aetiology, and control of sternal surgical site infections. J Hosp Infect. 85(3):206–12 (2013)

51. Coskun ,D., Aytac ,J., Aydinli ,A., Bayer ,A.: Mortality rate, length of stay and extra cost of sternal surgical site infections following coronary artery bypass grafting in a private medical centre in Turkey. J Hosp Infect. 60(2):176–9 (2005)

52. Ariyaratnam ,P., Bland ,M., Loubani ,M.: Risk factors and mortality associated with deep sternal wound infections following coronary bypass surgery with or without concomitant procedures in a UK population: a basis for a new risk model? Interact Cardiovasc Thorac Surg. 11(5):543–6 (2010)

53. Jenks ,P.J., Laurent ,M., McQuarry ,S., Watkins ,R.: Clinical and economic burden of surgical site infection (SSI) and predicted financial consequences of elimination of SSI from an English hospital. J Hosp Infect. 86(1):24–33 http://dx.doi.org/10.1016/j.jhin.2013.09.012 (2014)

## 
