# Supplementary material for: Economic Analysis of the European Healthcare Burden of Sternal-Wound Infections Following Coronary Artery Bypass Graft
Source: Front Public Health. 2020 Oct 23;8:557555. doi: 10.3389/fpubh.2020.557555 (PMC7645249; doi:10.3389/fpubh.2020.557555)
Supplement: Supplementary file 3 [file Table_3.docx]

Supplementary Material

# Supplementary table 3

| Country | Procedures | SWI events | SWI Burden | Mean cost per SWI | Burden per procedure |
| --- | --- | --- | --- | --- | --- |
| Name | N | N | Euro | Euro | Euro |
| Austria | 3,471 | 131 | 2,123,376 | 16,222 | 612 |
| Belgium | 7,142 | 244 | 3,444,875 | 14,095 | 482 |
| Czechia | 4,738 | 282 | 2,728,720 | 9,672 | 576 |
| Denmark | 3,694 | 154 | 3,291,684 | 21,321 | 891 |
| Estonia | 437 | 19 | 220,857 | 11,632 | 506 |
| Finland | 1,876 | 86 | 834,107 | 9,712 | 445 |
| France | 19,305 | 975 | 10,961,772 | 11,243 | 568 |
| Germany | 50,471 | 2594 | 31,669,649 | 12,209 | 627 |
| Greece | 5,422 | 485 | 5,174,612 | 10,675 | 954 |
| Hungary | 2,695 | 179 | 2,555,360 | 14,241 | 948 |
| Iceland | 195 | 14 | 211,692 | 4,979 | 1084 |
| Ireland | 926 | 37 | 776,175 | 21,071 | -838 |
| Italy | 20,942 | 1546 | 19,762,734 | 12,784 | 944 |
| Lithuania | 1,827 | 196 | 2,185,496 | 11,149 | 1196 |
| Malta | 171 | 4 | 54,410 | 12,794 | 319 |
| Netherlands | 9,690 | 815 | 16,686,727 | 20,478 | 1722 |
| Norway | 1,498 | 53 | 811,962 | 15,272 | 542 |
| Poland | 19,494 | 884 | 7,889,908 | 8,924 | 405 |
| Portugal | 3,646 | 294 | 2,897,670 | 9,842 | 795 |
| Romania | 4,514 | 300 | 2,704,612 | 9,012 | 599 |
| Serbia | 4,893 | 388 | 3,854,972 | 9,926 | 788 |
| Spain | 8,306 | 725 | 8,994,605 | 12,401 | 1083 |
| Sweden | 2,940 | 180 | 2,706,218 | 15,019 | 920 |
| Switzerland | 2,473 | 202 | 3,789,587 | 18,793 | -532 |
| Turkey | 67,710 | 2514 | 30,004,463 | 11,937 | 443 |
| UK | 16,546 | 359 | 4,457,720 | 12,402 | 269 |

**Supplementary Table 3.** Burden of CABG related SWIs by country with base parameters. Results rounded to the nearest full number
