# Supplementary material for: Economic Analysis of the European Healthcare Burden of Sternal-Wound Infections Following Coronary Artery Bypass Graft
Source: Front Public Health. 2020 Oct 23;8:557555. doi: 10.3389/fpubh.2020.557555 (PMC7645249; doi:10.3389/fpubh.2020.557555)
Supplement: Supplementary file 4 [file Table_4.docx]

Supplementary Material

# Supplementary table 4

| Country | Cost burden | | | ICU burden | | | GW burden | | | Readmission burden | | |
| --- | --- | --- | --- | --- | --- | --- | --- | --- | --- | --- | --- | --- |
| Name | Euro in millions | | | Care days | | | Care days | | | Events | | |
|  | Median | Min | Max | Median | Min | Max | Median | Min | Max | Median | Min | Max |
| Austria | 2.23 | 1.09 | 5.88 | 299 | 84 | 771 | 1,517 | 472 | 4,802 | 94 | 55 | 217 |
| Belgium | 4.31 | 3.14 | 6.85 | 595 | 356 | 1,030 | 3,486 | 2,262 | 6,794 | 195 | 147 | 292 |
| Czechia | 2.55 | 1.60 | 5.79 | 450 | 248 | 1,012 | 2,273 | 1,026 | 5,740 | 107 | 73 | 242 |
| Denmark | 2.86 | 1.78 | 5.53 | 361 | 210 | 645 | 1,899 | 946 | 3,523 | 103 | 64 | 175 |
| Estonia | 0.28 | 0.13 | 0.38 | 42 | 18 | 64 | 239 | 81 | 385 | 12 | 7 | 16 |
| Finland | 0.96 | 0.61 | 1.75 | 121 | 54 | 205 | 590 | 223 | 1,290 | 46 | 25 | 74 |
| France | 13.64 | 6.34 | 19.79 | 1,689 | 634 | 2,655 | 8,426 | 2,579 | 16,358 | 640 | 322 | 855 |
| Germany | 31.89 | 17.91 | 61.15 | 4,067 | 1,912 | 7,446 | 22,849 | 8,273 | 55,064 | 1,490 | 1,099 | 2,069 |
| Greece | 4.55 | 2.94 | 8.51 | 726 | 412 | 1,239 | 3,885 | 1,809 | 8,164 | 223 | 150 | 319 |
| Hungary | 2.57 | 1.60 | 4.81 | 561 | 411 | 1,056 | 2,995 | 1,785 | 6,120 | 81 | 60 | 113 |
| Iceland | 0.20 | 0.11 | 0.30 | 27 | 13 | 46 | 144 | 64 | 235 | 6 | 4 | 9 |
| Ireland | 0.76 | 0.36 | 1.13 | 99 | 55 | 158 | 580 | 286 | 961 | 25 | 10 | 34 |
| Italy | 16.63 | 9.86 | 26.57 | 2,361 | 1,359 | 3,940 | 13,366 | 6,516 | 25,196 | 726 | 476 | 1,199 |
| Lithuania | 1.90 | 1.20 | 3.95 | 286 | 185 | 510 | 1,567 | 744 | 3,492 | 87 | 65 | 123 |
| Malta | 0.08 | 0.03 | 0.14 | 15 | 6 | 28 | 75 | 24 | 182 | 3 | 2 | 5 |
| Netherlands | 9.86 | 5.49 | 13.74 | 1,699 | 773 | 2,768 | 9,251 | 3,348 | 13,375 | 262 | 169 | 367 |
| Norway | 1.06 | 0.71 | 1.78 | 104 | 63 | 181 | 556 | 248 | 947 | 37 | 23 | 58 |
| Poland | 11.35 | 7.56 | 18.69 | 1,516 | 705 | 2,479 | 7,368 | 3,211 | 12,760 | 660 | 446 | 970 |
| Portugal | 3.02 | 1.93 | 4.23 | 531 | 305 | 898 | 2,845 | 1,224 | 5,508 | 142 | 88 | 192 |
| Romania | 3.34 | 1.77 | 4.95 | 564 | 261 | 1,004 | 3,103 | 1,350 | 4,978 | 145 | 77 | 196 |
| Serbia | 3.65 | 2.23 | 5.22 | 580 | 298 | 919 | 3,237 | 1,482 | 5,383 | 190 | 133 | 318 |
| Spain | 7.66 | 5.23 | 13.53 | 1,114 | 718 | 2,278 | 6,150 | 3,496 | 11,982 | 303 | 220 | 475 |
| Sweden | 2.46 | 1.70 | 4.05 | 293 | 183 | 621 | 1,703 | 939 | 3,555 | 98 | 70 | 154 |
| Switzerland | 2.71 | 1.26 | 3.81 | 321 | 129 | 449 | 1,723 | 551 | 2,719 | 95 | 63 | 159 |
| Turkey | 43.70 | 27.87 | 61.95 | 6,543 | 2,849 | 8,879 | 35,610 | 13,789 | 53,869 | 2,121 | 1,141 | 3,130 |
| UK | 7.43 | 4.32 | 13.28 | 1,042 | 464 | 1,982 | 5,012 | 2,018 | 10,118 | 291 | 183 | 480 |

**Supplementary Table 4.** Median annual burden (range) of SWIs by country
